# Deoxysphingolipids and ether-linked diacylglycerols accumulate in the tissues of aged mice

**DOI:** 10.1186/s13578-019-0324-9

**Published:** 2019-08-05

**Authors:** Ayumi Ando, Masahiro Oka, Yoshinori Satomi

**Affiliations:** 1 Integrated Technology Research Laboratories, Takeda Pharmaceutical Company, Ltd., 26-1, Muraoka-Higashi 2-chome, Fujisawa, Kanagawa 251-8555 Japan; 2 Cardiovascular and Metabolic Drug Discovery Unit, Pharmaceutical Research Division, Takeda Pharmaceutical Company, Ltd., 26-1, Muraoka-Higashi 2-chome, Fujisawa, Kanagawa 251-8555 Japan

**Keywords:** Aging, Metabolomics, Lipidomics, Deoxysphingolipids, Ether-linked diacylglycerol

## Abstract

**Background:**

Senescence is a well-known risk factor for several diseases, such as neurodegenerative disorders. Therefore, studies exploring the mechanisms underlying aging are expected to guide the discovery of novel drug targets and biomarkers for these diseases. However, a comprehensive overview of the metabolic and lipidomic changes in healthy aging mammals is lacking. To understand the changes of metabolism with aging, especially lipid metabolism, we analyzed the metabolomes and lipidomes of the cerebral cortex, liver, femoral muscle, and epididymal fat in young and aged mice.

**Results:**

Two-dimensional cluster analysis revealed clear separation between the metabolite profiles of the aged and young groups. Deoxydihydroceramide (doxDHCer), deoxyceramide (doxCer), and ether-linked diacylglycerol (DAG) levels were elevated during aging.

**Conclusion:**

This is the first report of age-related variations in deoxysphingolipid and ether-linked DAG levels in mice. DoxCer, doxDHCer, and ether-linked DAGs may be associated with senescence in mammalian tissues.

**Electronic supplementary material:**

The online version of this article (10.1186/s13578-019-0324-9) contains supplementary material, which is available to authorized users.

## Dear Editor

Senescence underlies a wide range of diseases, such as cancer and neurodegenerative, cardiovascular, and metabolic diseases [1]. Studies clarifying the mechanisms of senescence are expected to guide the discovery of novel drug targets and biomarkers for these diseases. Understanding the molecular mechanisms of senescence could facilitate the identification of biomarkers of biological age. Furthermore, given the underlying multisystem network underlying the aging process, with changes occurring at molecular and organ-based levels, biological plausibility suggests that no single biomarker is likely to completely explain biological age.

Because multiple mechanisms contribute to the occurrence of senescence, molecular network analysis has been frequently used to comprehensively understand this process [1–3]. One such type is metabolomic analysis, which provides qualitative and quantitative information regarding all metabolites in a cell, tissue, or organ. Therefore, metabolomic studies have the potential to provide valuable insights into the mechanisms of senescence and senescence-related diseases. For instance, recent studies summarizing a combination of gene expression and metabolomic analyses have provided a footprint of aging in mice [3, 4]. These analyses demonstrated decreased long-chain acylcarnitine levels, increased free fatty acid levels, and marked reduction in various amino acid levels in the plasma of aged mice.

Additionally, research on the detection of accelerated aging in young adults using 18 biomarkers have enabled to note the differences in individuals with aging speed. This study has mentioned metabolic changes and physiological decline with aging by analyzing biomarkers [5]. However, the cause of physiological decline, i.e., senescence, with aging is still unclear. Furthermore, in addition to the less identified lipid- and disease-specific or single organ analysis, the lipidomics approach has been reported for aging and aging-related diseases [6, 7].

In this study, to clarify the changes in metabolism with aging, particularly the changes in lipid metabolism, we analyzed the metabolomes and lipidomes of the cerebral cortex, liver, femoral muscle, and epididymal fat in young (9 weeks old) and aged (114 weeks old) mice. A comprehensive analysis revealed previously unidentified metabolic changes that represented age-related variations in deoxydihydroceramide (doxDHCer), deoxyceramide (doxCer), and ether-linked diacylglycerol (DAG) levels in aged mice. These novel findings in the fields of molecular biology and senescence may provide further insight into aging-related diseases.

### Aged mice exhibited elevated levels of doxDHCer in epididymal fat

The total body weights and biochemical parameters of the young and aged mice were assessed in parallel to confirm the phenotyping data. Body weights, glucose, and total ketone body levels (see Additional file 1) were consistent with the results of a previous report comparing aged (22 months old) and young (3 months old) mice [3]. We used metabolomic and lipidomic techniques to analyze the cerebral cortex, liver, femoral muscle, and epididymal fat tissues in aged and young C57BL/6J mice (see Additional file 2) to understand the molecular mechanisms of senescence, and we identified more than 1000 metabolites in these tissues. The reasons for selecting these organs are as follows: (1) Femoral muscle and liver: these organs are the most energy-generating/consuming organs; therefore, we hypothesized that energy consumption affects aging; (2) Cerebral cortex: cognitive dysfunction is also known to be associated with aging, so we selected the cerebral cortex; and (3) Epididymal fat: Increase in body weight (especially fat) is associated with aging. However, no researcher has investigated the changes in body fat in association with these conditions. We hypothesized that increase in body fat is associated with the accumulation or generation of abnormal molecules in the fat tissue. Cardiolipins, ceramides, cholesterol esters, glycerolipids, dolichols, carnitines, ubiquinones, fatty acyl coenzyme A (CoA), free fatty acids, and ganglioside were identified by lipidomic analysis and metabolites from the tricarboxylic acid cycle, glycolysis, sugars, bile acids, pyrimidine pathway, nicotinate, and nicotinamide pathway amino acids and their metabolites were identified by metabolomic analysis (see Additional file 3). Then, the profiles of aged and young mice were compared, and cluster analysis revealed clear differences in terms of these profiles between these two groups. However, it was difficult to visualize the results because numerous metabolites were present in the results of cluster analysis. Therefore, we performed re-analysis to focus on deoxysphingolipids (doxSLs) and ether-linked DAGs. We could not find any report regarding the relationship between aging and these lipids at the time of data analyses. Thus, we chose doxSLs and ether-linked DAGs as focused metabolites. They were chosen as unique metabolites in aging for subsequent analyses.

**Fig. 1:**
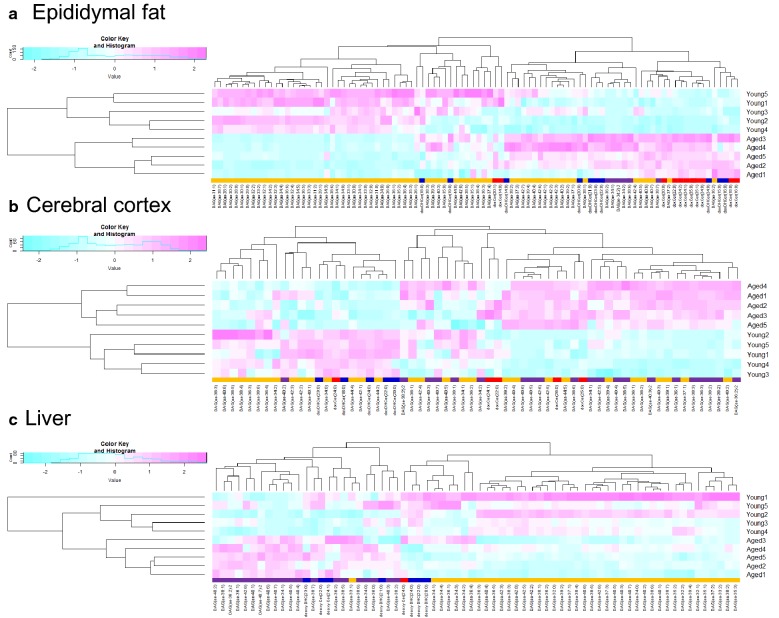
Two-dimensional cluster heat-map visualization of detected lipids and metabolites from aged and young mice. DoxCers (red), doxDHCers (blue), DAG (aa) (yellow) and DAG(ae) (purple) in epididymal fat (**a**), the cerebral cortex (**b**), and the liver (**c**) in young (9 weeks old) and aged (114 weeks old) mice. DAG, diacylglycerol; aa, di-acyl; ae, acyl and ether; doxDHCer, deoxydihydroceramide; doxCer, deoxyceramide

**Fig. 2:**
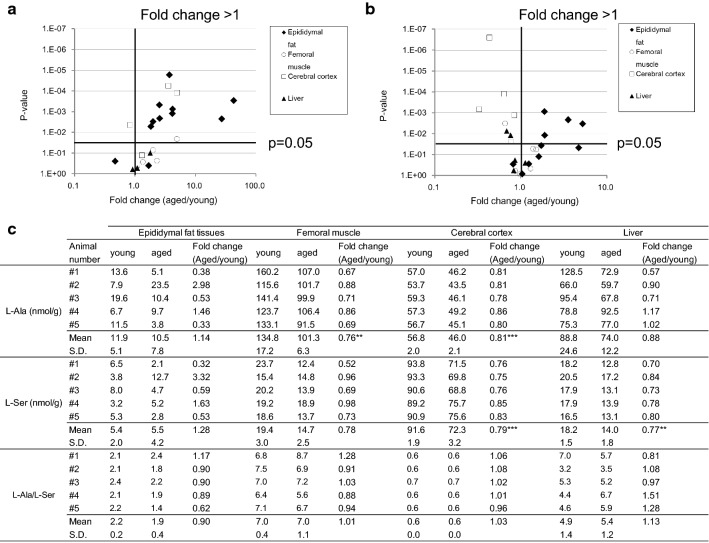
Volcano plot of the fold changes in the levels of doxSLs and concentrations of l-Ser and l-Ala. DoxCer (**a**) and doxDHCer (**b**) between aged and young mice and the corresponding p-values (white square: cerebral cortex; black triangle: liver; white circle: femoral muscle; black diamond: epididymal fat tissues). Each data point represents the mean value of five mice per group and different carbon chain-length and degree of unsaturation of fatty acid in each metabolite. A log 10 scale is used for the x-axis. **c** Fold changes (aged/young) in the mean concentrations of l-Ser and l-Ala. P-values are indicated for the metabolites with significant differences in abundance between groups. ***P < 0.001, **P < 0.01, *P < 0.05

From cluster analysis, we also identified unique molecular species of ether-linked DAGs and sphingolipids in aged mice that varied from the ceramide species generally found in the epididymal fat tissues, cerebral cortex, and liver. We observed higher ether-linked DAG levels in the cerebral cortex, fat tissue, and liver of the aged mice than in equivalent tissues of young mice (Figs. 1a–c). The femoral muscle showed no significant changes (see Additional file 4). Furthermore, the doxCer levels were significantly higher (P < 0.05, fold change > 1) in the epididymal fat tissues (Fig. 2a) and cerebral cortex of aged mice than in the equivalent tissues in young ones. Moreover, doxDHCer levels were significantly higher in the epididymal fat tissues (Fig. 2b). These doxSLs have been identified in the plasma of patients with hereditary sensory and autonomic neuropathy [8].

The levels of two types of doxSL, i.e., doxCer and doxDHCer, were significantly higher in the epididymal fat tissue of aged mice than those in young ones (see Additional files 5 and 6). Conversely, in the cerebral cortex, doxCer levels were higher but doxDHCer levels were lower in aged mice than those in young ones. Further, significant differences were not observed in the levels of these molecules in the liver or femoral muscle. These molecules are synthesized by serine palmitoyltransferase (SPT), with the preferred substrates being serine and palmitoyl-CoA. SPT catalyzes the condensation of l-serine (l-Ser) and palmitoyl-CoA to form 3-keto-sphinganine and ultimately sphinganine. Sphinganine is subsequently N-conjugated with a second fatty acid to form dihydroceramide. Moreover, SPT can use l-alanine (l-Ala), as a substitute, and conjugated alanine forms doxSLs [9] via 1-deoxysphinganine and 1-deoxysphingosine. In an in vitro study, doxSLs, such as doxCer and doxDHCer, were generated in the presence of high l-Ala/l-Ser ratios in the lipid droplets in cells [9]. Based on this information, we analyzed l-Ser and l-Ala levels in several tissues to clarify the reason for their accumulation in epididymal fat.

### Epididymal fat tissues displayed no changes in l-Ser and l-Ala levels

We analyzed l-Ser and l-Ala levels in the liver, cerebral cortex, epididymal fat, and femoral muscle tissue in aged and young mice to determine whether the l-Ala/l-Ser ratio changed with aging via amino acid quantification (Fig. 2c). The aged mice exhibited lower l-Ser levels in the liver and cerebral cortex as well as lower l-Ala in the cerebral cortex and femoral muscle than those in young ones. Conversely, no changes were observed in the epididymal fat. Nevertheless, lower l-Ser and l-Ala levels and higher doxCer and dosDHCer levels in the epididymal fat than those in the other tissues were observed. There were no changes between aged and young mice with respect to the l-Ala/l-Ser ratio, which was 0.9–1.13. In addition, the standard deviation of the l-Ala/l-Ser ratio was lower than the individual values of l-Ala and l-Ser in all tissues. We hypothesize that these phenomena are attributable to the accumulation of doxCers and doxDHCers in epididymal fat rather than de novo synthesis because we did not observe differences in l-Ser levels and observed lower l-Ser levels in epididymal fat than in the other tissues.

In the cerebral cortex, significantly lower l-Ser and l-Ala levels were observed in aged mice than in young ones (Fig. 2c). A previous report found that the l-Ala/l-Ser ratio is a likely determinant of doxSL production; however, l-Ser levels were significant reduced in the cerebral cortex. Regarding the decreases in doxDHCer levels in the cerebral cortex, we speculate that there are two reasons for this: First, the lower l-Ala and l-Ser levels in the aged group than in the young group may have reduced the total production of doxDHCer. Second, the suppression of the enzyme activity, including serine palmitoyltransferase, 3-dehydrosphinganine reductase, and ceramide synthetase in the ceramide de novo synthesis until doxDHCer in the aged group, may also affect the total production of doxDHCer. Regarding the increase in doxCer levels in the cerebral cortex of the aged group, we speculate that the activation of the sphingolipid salvage pathway is one possible cause of the increase in doxCer levels. Because the decreases in doxDHCer levels had already been observed, we speculate that doxCer synthesis from the de novo synthesis pathway is less likely. The use of enzyme activity to evaluate the decrease in doxDHCer levels in the brains of aged mice may be a useful method in understanding this phenomenon.

Recently, some reports have described an association between doxSLs and diseases; for example, patients with aberrant plasma l-Ser and l-Ala levels, a situation which can be secondary to mitochondrial disorders, exhibit peripheral neuropathy with similar elevated levels of atypical sphingolipids [10]. Further, doxSL levels are elevated in type 2 diabetes mellitus [11], and such compounds are cytotoxic for insulin-producing cells [12]. These symptoms are similar to those of aging-associated diseases that are caused by the progressive loss or dysfunction of cells. Based on the findings of our study and those from the abovementioned reports, we believe that further studies are warranted to understand age-related diseases and doxSLs. These studies indicate that the intracellular metabolites of deoxysphinganine, particularly doxDHCer, are important cytotoxic mediators [12, 13]. To our knowledge, the present study is the first reporting age-related variations in doxCer and doxDHCer levels in vivo.

### Increase in ether-linked DAG levels in aged tissues

**Fig. 3:**
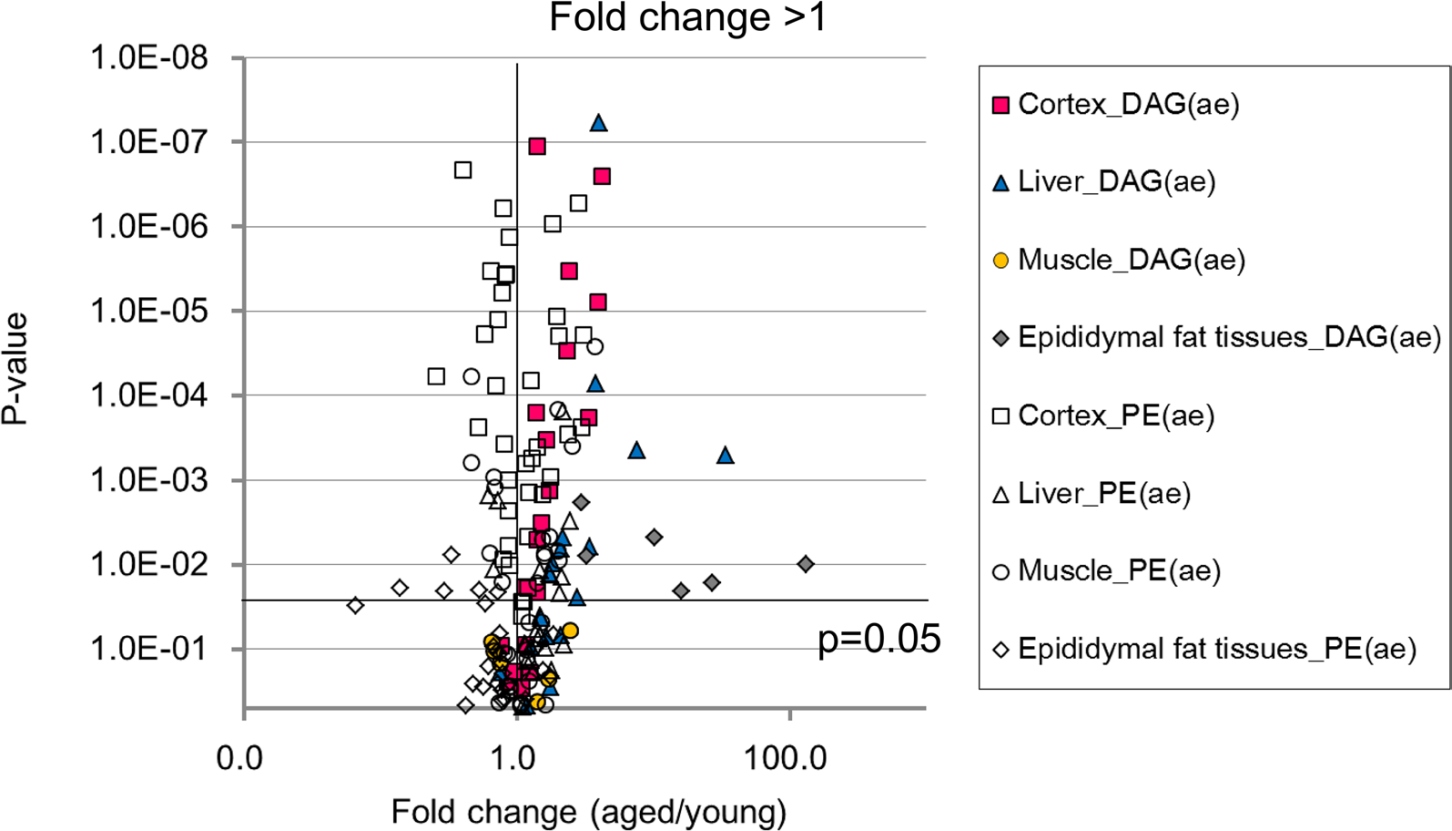
Volcano plot of the fold changes in the levels of ether-linked diacylglycerols and ethanolamine plasmalogens. Ether-linked DAG (pink square: cerebral cortex; blue triangle: liver; yellow circle: femoral muscle; gray diamond: epididymal fat tissue) and ethanolamine plasmalogens (white square: cerebral cortex; white triangle: liver; white circle: femoral muscle; white diamond: epididymal fat tissues) between aged and young mice and the corresponding p-values. Each data point represents the mean value of five mice per group and different carbon chain-length and degree of unsaturation of fatty acid in each metabolite. A log 10 scale is used for the x-axis

We found that ether-linked DAG levels were significantly higher (P < 0.05, fold change > 1) in the epididymal fat tissues, cerebral cortex, and liver of aged mice than the equivalent tissues in young ones (Fig. 3). However, the detailed biosynthetic pathway for ether-linked DAGs has not been determined in aged mammals.

The first step in the synthesis of ether–lipids occurs in peroxisomes. Most phospholipids contain a hydrophobic fatty acid and hydrophilic phosphoglycerol head group. Conversely, ether-linked phospholipids contain long-chain fatty alcohols connected by an ether bond to the phosphoglycerol structure [14]. Plasmalogen and platelet-activating factor (PAF) are the members of the latter group of lipid structures, with ethanolamine or choline being bonded as a head group to a fatty acid ether that is bonded to a fatty acid ester, which is in turn bonded to a glycerol skeleton [15]. Furthermore, PAF concentration is lower than that of ethanolamine plasmalogens [14–17]. Therefore, we hypothesized that the observed decrease in ethanolamine plasmalogen levels is caused by the elimination of this head group.

Analysis of the fold change in ethanolamine plasmalogen levels in aged and young mice (Fig. 3) revealed a significant decrease in its levels in aged mice, particularly in the cerebral cortex and epididymal fat tissues, and this decrease was accompanied by an increase in ether-linked DAG levels. Ether-linked diglyceride species, in contrast to ester-linked diglyceride species, do not activate protein kinase C [18] and ether-linked DAGs can induce growth arrest, but not apoptosis, in vascular/aortic cell lines [19].

The mechanisms underlying the accumulation and biosynthesis of ether-linked DAGs in aged mammals are unknown. The lipid profile observed in our analysis predicts that ethanolamine plasmalogens are the substrates of ether-linked DAGs.

Plasmalogen levels in the brain are reduced in patients with Alzheimer’s disease [20] and those with neurological injuries, including ischemia and spinal cord trauma [21]. A previous report suggested that the stimulation of plasmalogen-selective phospholipase A2 (PSPLA2) caused this phenomenon, and plasmalogen degradation by PSPLA2 resulted in the generation of arachidonic acid, eicosanoids, and PAFs [21]. In the present study, plasmalogen levels reduced in the cerebral cortex, whereas ether-linked DAG levels increased. Moreover, ether–lipid levels are reportedly elevated in human tumors, making such tumors more aggressive [22]. A recent study has evaluated the alkylglycerone phosphate synthetase enzyme activities promotes tumor growth, which in the peroxisomally localized activities promotes tumor growth [22]. Furthermore, the investigation of the relationship of growth arrest and apoptosis with ether-linked DAG levels may provide insights into healthy aging.

We hypothesize that ethanolamine plasmalogen hydrolysis increases ether-linked DAG levels in the cerebral cortex and epididymal fat tissues. Ether-linked DAGs have been identified in mammalian tissues such as the mouse brain and heart [23]; however, quantitative changes in its levels in aged animal models have not yet been reported. Therefore, further studies on the biological functions of ether-linked DAGs are warranted to determine whether these molecules play a role in the aging process.

We identified doxDHCer, doxCer, and ether-linked DAGs as aging-associated lipids, and their possible role in senescence could be investigated. Although some metabolic changes associated with aging have previously been investigated, to the best of our knowledge, this is the first time that changes in the levels of these three lipids have been unambiguously demonstrated using mass spectrometry. However, further research is needed to fully establish the relevance of these lipids to aging and clarify the biosynthesis of the newly identified age-related lipids. In addition, two questions need to be answered in future studies. First, what is the significance of the decrease in doxDHCer levels in the cerebral cortex? Second, can doxDHCer, doxCer, and ether-linked DAG levels be detected in the plasma of aged mammals? In this study, we could not conduct lipidomic analysis of plasma because of low sample amounts. If the plasma levels of doxDHCer, doxCer, and ether-linked DAGs can be detected, these data may provide insights into healthy senescence and facilitate the discovery of novel drug targets and biomarkers. The results obtained in this study will contribute to the understanding of the aging process and the prevention of accelerated aging and aging-associated diseases.

## Additional files

**Additional file 1.** Plasma biochemical parameters. Glucose (Glu), triglyceride (TG), glutamic pyruvic transaminase (GPT), total cholesterol (T-CHO), and total ketone body (T-KB) levels; body weights; and weight reduction rates (%).

**Additional file 2.** Materials and methods.

**Additional file 3.** A list of identified metabolites and lipids in the cerebral cortex, liver, femoral muscle, and epididymal fat in aged and young mice. Retention time (RT), observed mass (ObsMass), coefficient of variation of mixQC (CV), and ion from lipidomics analysis (Ion) and database identifiers (KEGG and HMDB) for metabolomics analysis are also included in table. Abbreviations: MG, monoacyl; DG, diacylglycerol; TG, triacylglycerol; aa, di-acyl; aaa, tri-acyl; aaaa, tetra-acyl; ae, acyl and ether; phosphatidylcholine, PC; lysophosphatidylcholine, LPC; phosphatidylethanolamine, PE; lysophosphatidylethanolamine, LPE; phosphatidylserine, PS; lysophosphatidylserine, LPS; phosphatidylglycerol, PG; lysophosphatidylglycerol, LPG; phosphatidic acid, PA; lysophosphatidic acid, LPA; phosphatidylinositol, PI; lysophosphatidylinositol, LPI; CL, cardiolipins; Cer, ceramides; SM, sphingomyelins.

**Additional file 4.** Ether-linked DAGs in epididymal fat (a), the cerebral cortex (b), liver (c), and femoral muscle (d) in young (9 weeks old) and aged (114 weeks old) mice. Data are presented as box-and-whisker plot, with whiskers showing minimum and maximum values and each dot plot showing individual values (white bar and blue dot: young, gray bar and red dot: aged). P-values are indicated for the metabolites with significant differences in terms of abundance between groups. ***P < 0.001, **P < 0.01, *P < 0.05.

**Additional file 5.** The doxCers in epididymal fat (a), the cerebral cortex (b), liver (c), and femoral muscle (d) in young (9 weeks old) and aged (114 weeks old) mice. Data are presented as box-and-whisker plot, with whiskers showing minimum and maximum values and each dot plot showing individual values (white bar and blue dot: young, gray bar and red dot: aged). P-values are indicated for the metabolites with significant differences in terms of abundance between groups. Abbreviations: DG, diacylglycerol; ae, acyl and ether. ***P < 0.001, **P < 0.01, *P < 0.05.

**Additional file 6.** The doxDHCers in epididymal fat (a), the cerebral cortex (b), liver (c), and femoral muscle (d) in young (9 weeks old) and aged (114 weeks old) mice. Data are presented as box-and-whisker plot, with whiskers showing minimum and maximum values and each dot plot showing individual values (white bar and blue dot: young, gray bar and red dot: aged). P-values are indicated for the metabolites with significant differences in terms of abundance between groups. ***P < 0.001, **P < 0.01, *P < 0.05.

## Data Availability

The Materials and methods for this study are documented in detail in Additional file 2. The metabolomics and metadata reported in this paper are available via http://www.metabolomicsworkbench.org/. (Data track IDs, 1510, 1521, 1523, and 1525).

## Abbreviations

doxDHCer: deoxydihydroceramide
doxCer: deoxyceramide
DAG: diacylglycerol
CoA: coenzyme A
SPT: serine palmitoyltransferase
PSPLA2: plasmalogen-selective phospholipase A2
l-Ser: l-serine
l-Ala: l-alanine
PAFs: platelet-activating factors

## References

[1] López-Otín C, Blasco MA, Partridge L, Serrano M, Kroemer G (2013). The hallmarks of aging. Cell.

[2] Petrosillo G, Matera M, Casanova G, Ruggiero FMM, Paradies G (2008). Mitochondrial dysfunction in rat brain with aging Involvement of complex I, reactive oxygen species and cardiolipin. Neurochem Int.

[3] Houtkooper RH, Argmann C, Houten SM, Cantó C, Jeninga EH, Andreux PA (2011). The metabolic footprint of aging in mice. Sci Rep..

[4] Seo C, Hwang YH, Kim Y, Joo BS, Yee ST, Kim CM (2016). Metabolomic study of aging in mouse plasma by gas chromatography–mass spectrometry. J Chromatogr B Analyt Technol Biomed Life Sci..

[5] Belsky DW, Caspi A, Houts R, Cohen HJ, Corcoran DL, Danese A (2015). Quantification of biological aging in young adults. Proc Natl Acad Sci USA.

[6] Tu J, Yin Y, Xu M, Wang R, Zhu ZJ (2017). Absolute quantitative lipidomics reveals lipidome-wide alterations in aging brain. Metabolomics.

[7] Braun F, Rinschen MM, Bartels V, Frommolt P, Habermann B, Hoeijmakers JHJ (2016). Altered lipid metabolism in the aging kidney identified by three layered omic analysis. Aging.

[8] Penno A, Reilly MM, Houlden H, Laurá M, Rentsch K, Niederkofler V (2010). Hereditary sensory neuropathy type 1 is caused by the accumulation of two neurotoxic sphingolipids. J Biol Chem.

[9] Esaki K, Sayano T, Sonoda C, Akagi T, Suzuki T, Ogawa T (2015). l-Serine deficiency elicits intracellular accumulation of cytotoxic deoxysphingolipids and lipid body formation. J Biol Chem.

[10] Ferreira CR, Goorden SMI, Soldatos A, Byers HM, van der Ghauharali-Vlugt JMM, Beers-Stet FS (2018). Deoxysphingolipid precursors indicate abnormal sphingolipid metabolism in individuals with primary and secondary disturbances of serine availability. Mol Genet Metab..

[11] Bertea M, Rütti MF, Othman A, Marti-Jaun J, Hersberger M, von Eckardstein A (2010). Deoxysphingoid bases as plasma markers in diabetes mellitus. Lipids Health Dis..

[12] Zuellig RA, Hornemann T, Othman A, Hehl AB, Bode H, Guntert T (2014). Deoxysphingolipids, novel biomarkers for type 2 diabetes, are cytotoxic for insulin-producing cells. Diabetes.

[13] Kramer R, Bielawski J, Kistner-Griffin E, Othman A, Alecu I, Ernst D (2015). Neurotoxic 1-deoxysphingolipids and paclitaxel-induced peripheral neuropathy. FASEB J..

[14] Shevchenko A, Simons K (2010). Lipidomics: coming to grips with lipid diversity. Nat Rev Mol Cell Biol.

[15] Nagan N, Zoeller RA (2001). Plasmalogens: biosynthesis and functions. Prog Lipid Res.

[16] Latchoumycandane C, Nagy LE, McIntyre TM (2015). Myeloperoxidase formation of PAF receptor ligands induces PAF receptor-dependent kidney injury during ethanol consumption. Free Radic Biol Med..

[17] Otoki Y, Kato S, Kimura F, Furukawa K, Yamashita S, Arai H (2017). Accurate quantitation of choline and ethanolamine plasmalogen molecular species in human plasma by liquid chromatography–tandem mass spectrometry. J Pharm Biomed Anal.

[18] Musial A, Mandal A, Coroneos E, Kester M (1995). Interleukin-1 and endothelin stimulate distinct species of diglycerides that differentially regulate protein kinase C in mesangial cells. J Biol Chem.

[19] Houck KL, Fox TE, Sandirasegarane L, Kester M (2008). Ether-linked diglycerides inhibit vascular smooth muscle cell growth via decreased MAPK and PI3K/Akt signaling. Am J Physiol Heart Circ Physiol..

[20] Ginsberg L, Rafique S, Xuereb JH, Rapoport SI, Gershfeld NL (1995). Disease and anatomic specificity of ethanolamine plasmalogen deficiency in Alzheimer’s disease brain. Brain Res.

[21] Farooqui AA, Horrocks LA (2001). Plasmalogens: workhorse lipids of membranes in normal and injured neurons and glia. Neuroscientist..

[22] Benjamin DI, Cozzo A, Ji X, Roberts LS, Louie SM, Mulvihill MM (2013). Ether lipid generating enzyme AGPS alters the balance of structural and signaling lipids to fuel cancer pathogenicity. Proc Natl Acad Sci USA..

[23] Yang K, Jenkins CM, Dilthey B, Gross RW (2015). Multidimensional mass spectrometry-based shotgun lipidomics analysis of vinyl ether diglycerides. Anal Bioanal Chem.

