# Supplementary material for: Deoxysphingolipids and ether-linked diacylglycerols accumulate in the tissues of aged mice
Source: Cell Biosci. 2019 Aug 5;9:61. doi: 10.1186/s13578-019-0324-9 (PMC6683348; doi:10.1186/s13578-019-0324-9)
Supplement: Supplementary file 2 — Additional file 2. Materials and methods. [file 13578_2019_324_MOESM2_ESM.docx]

# Material and methods

## *Reagents*

Acetonitrile, isopropanol, methanol, acetic acid, formic acid (all of liquid chromatography–mass spectrometry [LC/MS] grade), octylamine, 2 M ammonia isopropanol solution, 0.2 M disodium dihydrogen ethylenediaminetetraacetic acid (EDTA-2Na) solution, and pyridine were purchased from Wako Pure Chemicals (Osaka, Japan). The amino acid standard solution was purchased from Agilent Technologies Inc. (Santa Clara, CA, USA). Methoxyamine hydrochloride; N,O-bis(trimethylsilyl)trifluoroacetamide (BSTFA) and trimethylchlorosilane (99:1) mixed in a solvent (Sylon BFT); algal amino acid mixture (U-^13^C); and N-tert-butyldimethylsilyl-N-methyltrifluoroacetamide with 1% tert-butyldimethylchlorosilane (MTBSTFA with 1% t-BDMCS) were purchased from Sigma-Aldrich (St. Louis, MO, USA).

## *Animals*

Seven-week-old C57BL/6J male mice were purchased from CLEA (Tokyo, Japan) and housed in groups of four in a temperature-controlled facility with a 12-h/12-h light/dark cycle. Male mice belonging to the aged group received standard chow (CE-2, CLEA) and water ad libitum until 114 weeks of age. Under the same conditions, young mice were acclimatized for 14 days till 9 weeks of age. All mice were weighed before the day of investigation and before the start of the experiment. Considering that food intake can affect metabolomic data, all mice were reweighed and decapitated after overnight fasting (16 h) for sample collection. Tissue collection was conducted within 3 min to avoid postmortem degradation; the collected tissues were placed in 2.0 mL test tubes, immediately frozen in liquid nitrogen, and weighed. For plasma preparation, approximately 200 μL of blood following decapitation was directly collected into tubes containing 10 μL EDTA-2Na (150 mM, pH 7.4) on ice, followed by immediate centrifugation (10,000 ×*g*, 10 min, 4°C). The collected plasma was only used for biochemical analysis owing to insufficient sample amounts. All samples were stored at −80°C until use. All animal experiments were conducted in accordance with the protocol reviewed and approved by the Institutional Animal Care and Use of Takeda Pharmaceutical Co., Ltd.

## *Biochemistry*

Plasma glutamic pyruvic transaminase activity and glucose, total ketone body, total cholesterol, and triglyceride concentrations were measured via enzymatic methods using a Hitachi 7180 biochemical autoanalyzer (Hitachi High-Technologies Corp., Tokyo, Japan).

## *Global metabolomic analysis*

### 1 Sample preparation

Global metabolomic profiles were obtained by combining the data derived from the following platforms: LC/MS/MS (for lipidomics), gas chromatography/MS, amino acid quantification, and ion-pair (IP)-LC/MS/MS.

Tissues from aged (n = 5) and young mice (n = 5) were homogenized in isopropanol (100 mg/mL) for lipidomics and in methanol (100 mg/mL) for GC/MS, amino acid quantification, and IP-LC/MS/MS using a ShakeMaster Auto instrument (BioMedical Science, Tokyo, Japan). Homogenization was followed by centrifugation at 15,000 rpm for 5 min. During these processes, the samples were stored in ice-cold tubes. The mixed quality control (mixQC) samples were prepared by mixing aliquots of each tissue homogenate, followed by centrifugation as described previously. MixQC samples were measured for every two samples, and precision, given as the coefficient of variation, was calculated using five-times repeated analysis of mixQC data. Metabolites and lipids with a coefficient of variation of less than 30% were adopted.

### 2 Lipidomics

From aged (n = 5) and young mice (n = 5), 5 μl of samples (supernatants from the isopropanol homogenates) were injected into an XBridge C18 column (2.1 × 50 mm, 3 μm; Waters, Milford, CT, USA). Chromatographic separation was performed on an Ultimate3000 RSLC LC/MS system (Thermo Fisher Scientific Inc., Sunnyvale, CA, USA) using a gradient elution comprising mobile phases A (0.01% acetic acid and 1 mM ammonia in MilliQ water) and B (0.001% acetic acid and 0.2 mM ammonia in ethanol/isopropanol [3:1]) at a flow rate of 0.7 mL/min. The following gradient program was used: 0–2 min, 2% B; 2–10 min, 2%–100% B; 10–12 min, 100% B; and 12–15 min, 2% B. The analysis was performed using an Orbitrap XL mass spectrometer (Thermo Fisher Scientific Inc., San Jose, CA, USA) operated at 60,000 full width at half maximum in the positive and negative ionization modes. Raw LC/MS data were processed to align the retention time and eliminate chemical noise using the Expressionist Refiner MS software, version 8.2 (Genedata AG, Basel, Switzerland). Further, lipids were identified based on their molecular mass and retention time using an HPLC chromatograph [1]. The data including monoisotopic m/z value and retention time information were exported, and further processing was performed using Excel.

### 3 IP-LC/MS/MS

### The LC/MS/MS system comprised a Nexera HPLC system (Shimadzu Co., Kyoto, Japan) and 5500 QTRAP mass spectrometer (AB Sciex Pte., Ltd., Toronto, Canada). From aged (n = 5) and young mice (n = 5), 10 μl of samples (supernatants from the methanol homogenates) were injected into an Atlantis T3 reversed-phase column (2.1 × 100 mm, 3 μm, 130 Å; Waters) maintained at 35°C, and chromatographic separation was performed using a gradient elution (0–2 min, 1% B; 2–12.5 min, 1%–100% B; 12.5–15 min, 100% B; and 15–27 min, 1% B) comprising mobile phases A (0.1% octylamine, 0.07% acetic acid and 10 μM EDTA-2Na in MilliQ water) and B (0.07% acetic acid in methanol/isopropanol [4:1]). All target molecules were observed in the multiple reaction monitoring (MRM) mode with simultaneous polarity switching. The MRM conditions have been previously described [2]. The LC/MS/MS data were processed using the MultiQuant software (AB Sciex Pte., Ltd., Toronto, Canada), and further processing was performed using Microsoft Excel.

### 4 GC/MS

From aged (n = 5) and young mice (n = 5), 50 μl of samples (supernatants from the methanol homogenates) were dried under a nitrogen stream. The dehydrated supernatant was then subjected to a derivatization reaction by adding 50 μL of methoxyamine hydrochloride in pyridine (15 mg/mL) and incubating the solution at 30°C for 30 min. This step was followed by a second derivatization reaction, in which 100 μL of trimethylsilylation reagent was directly added to the first reaction mixture and incubated at 60°C for 30 min. The reaction mixture (1 μL) was injected into an Agilent 7890A GC system for chromatography in the splitless injection mode using a GC Injector 80 autosampler (Agilent Technologies). GC separation was performed on a J&W Scientific HP-5MS-DG column (30 m × 0.25 mm × 0.25 μm; Agilent Technologies) using a temperature gradient, with the temperature increasing from 60°C to 325°C at 10°C/min, and a consistent helium gas flow of 1 mL/min. The eluate was ionized via electron impact ionization (70 eV) with an ion source temperature of 280°C and introduced into an Agilent 5975C mass spectrometer. Each target molecule was detected in the full-scan mode, and metabolite annotation was performed using GC/MSD ChemStation Agilent Quantitative Software (Agilent Technologies) with Agilent Fiehn Metabolomics RTL Library (Agilent Technologies). The data including the name and retention time of each metabolite were exported, and further processing was conducted using Microsoft Excel.

### 5 Amino acid quantification

For amino acid quantification, tert-butyldimethylsilyl (tBDMS) derivatization for GC/MS analysis was performed. For sample preparation, supernatants (50 μL) from the methanol homogenates (five mice each from the young and aged groups) were mixed with 10 μL of an algal amino acid mixture (U-^13^C), which served as the internal standard (IS), in a glass vial and dried under a nitrogen stream. Next, the dehydrated supernatant was subjected to a derivatization reaction by adding 50 μL of a 1:1 solution of MTBSTFA (with 1% t-BDMCS) and acetonitrile. The glass vial was tightly capped and incubated at 70°C for 180 min. Finally, 10 μL of the derivatized solution was used for GC/MS analysis.

Calibration standards were prepared by diluting the standard stock solution in methanol at final concentrations of 0.1, 1, 10, 25, 50, 100, and 125 nmol/mL for all amino acids and 0.4 mg/mL for IS. Methanol (50 µL) was added to a glass tube with 10 μL of IS solution, and derivatization was performed as described previously. The calibration curve was prepared using the least-squares linear regression method with 1/*x* weighting.

## *Statistical analysis*

The fold changes in metabolite levels between the aged and young groups were calculated as the ratios of the peak area values of each metabolite using integrated data. Raw mass spectrometric data from IP-LC/MS/MS and lipidomics were analyzed using “R” (<http://cran.r-project.org/>) for two-dimensional cluster analysis and heat-map visualization after *z* score-standardized (mean 0 [SD 1]). The R analysis code is provided at <https://github.com/Ayumi0Ando/Rcode_agedmice>. The data shown in the figures are presented as volcano plots, with fold changes indicated using peak areas and *p*-values and outliers represented as small symbols. The data shown as box-and-whisker plots were calculated using GraphPad Prism version 7.00 (GraphPad Software, Inc.). The whiskers of box plots represented Min to Max. Welch’s *t*-tests were performed for statistical comparisons between young and old mice, and *p* < 0.05 was considered statistically significant.

## *References*

1. Satomi Y, Hirayama M, Kobayashi H. One-step lipid extraction for plasma lipidomics analysis by liquid chromatography mass spectrometry. J Chromatogr B Analyt Technol Biomed Life Sci. 2017;1063:93-100.

2. Yuan M, Breitkopf SB, Yang X, Asara JM. A positive/negative ion-switching, targeted mass spectrometry-based metabolomics platform for bodily fluids, cells, and fresh and fixed tissue. Nat Protoc. 2012;7:872-81.
