# Supplementary figures and images for: Deoxysphingolipids and ether-linked diacylglycerols accumulate in the tissues of aged mice
Source: Cell Biosci. 2019 Aug 5;9:61. doi: 10.1186/s13578-019-0324-9 (PMC6683348; doi:10.1186/s13578-019-0324-9)

## Slide 1
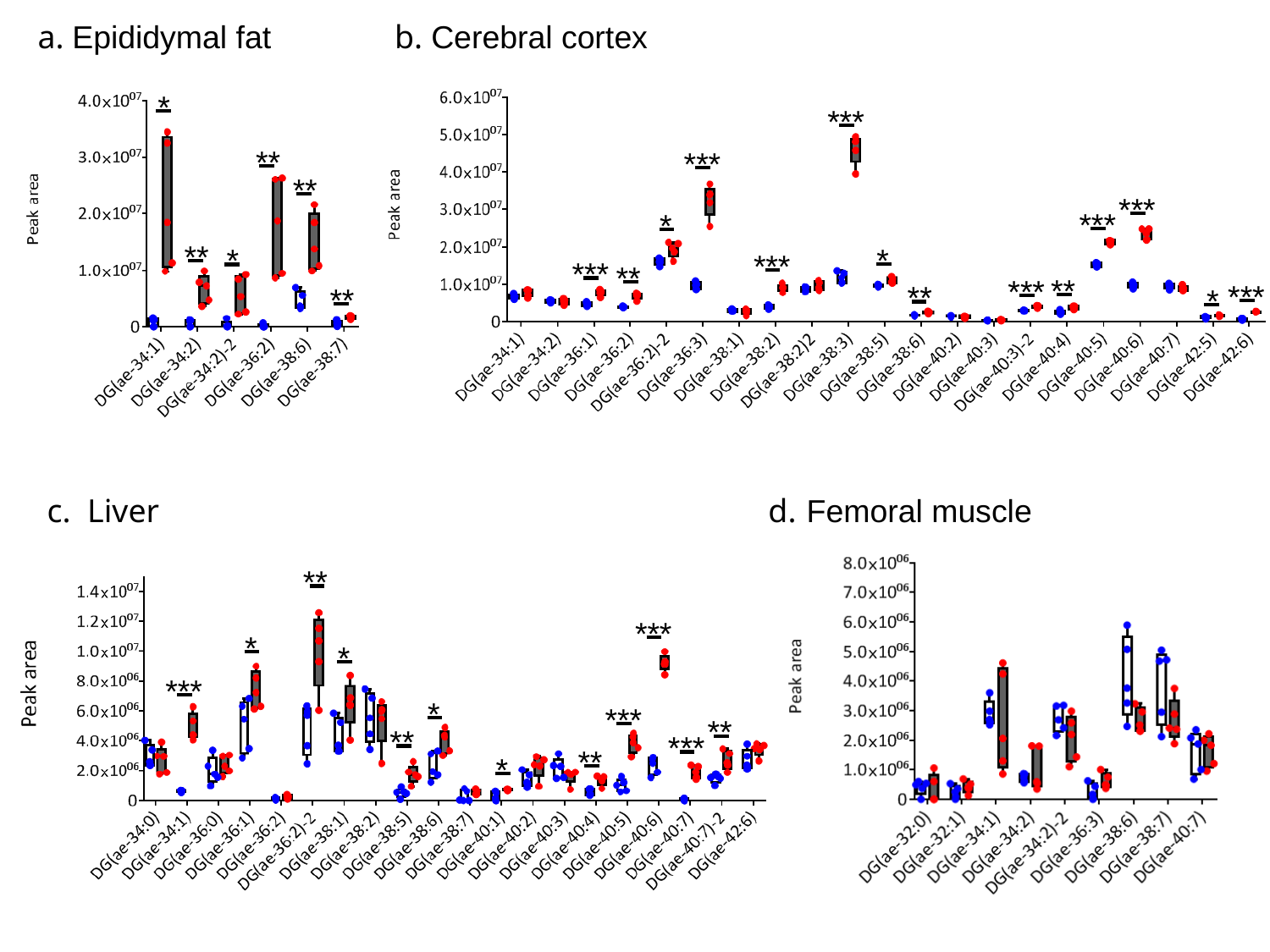

a. Epididymal fat b. Cerebral cortex
*
***
**
***
**
***
***
*
**
*
*
***
***
**
**
***
***
**
**
*
c. Liver d. Femoral muscle
**
***
*
*
***
*
***
**
**
***
**
*

Supplement: Supplementary file 4 — Additional file 4. Ether-linked DAGs in epididymal fat (a), the cerebral cortex (b), liver (c), and femoral muscle (d) in young (9 weeks old) and aged (114 weeks old) mice. Data are presented as box-and-whisker plot, with whiskers showing minimum and maximum values and each dot plot showing individual values (white bar and blue dot: young, gray bar and red dot: aged). P-values are indicated for the metabolites with significant differences in terms of abundance between groups. ***P < 0.001, **P < 0.01, *P < 0.05. [file 13578_2019_324_MOESM4_ESM.pptx]

## Slide 1
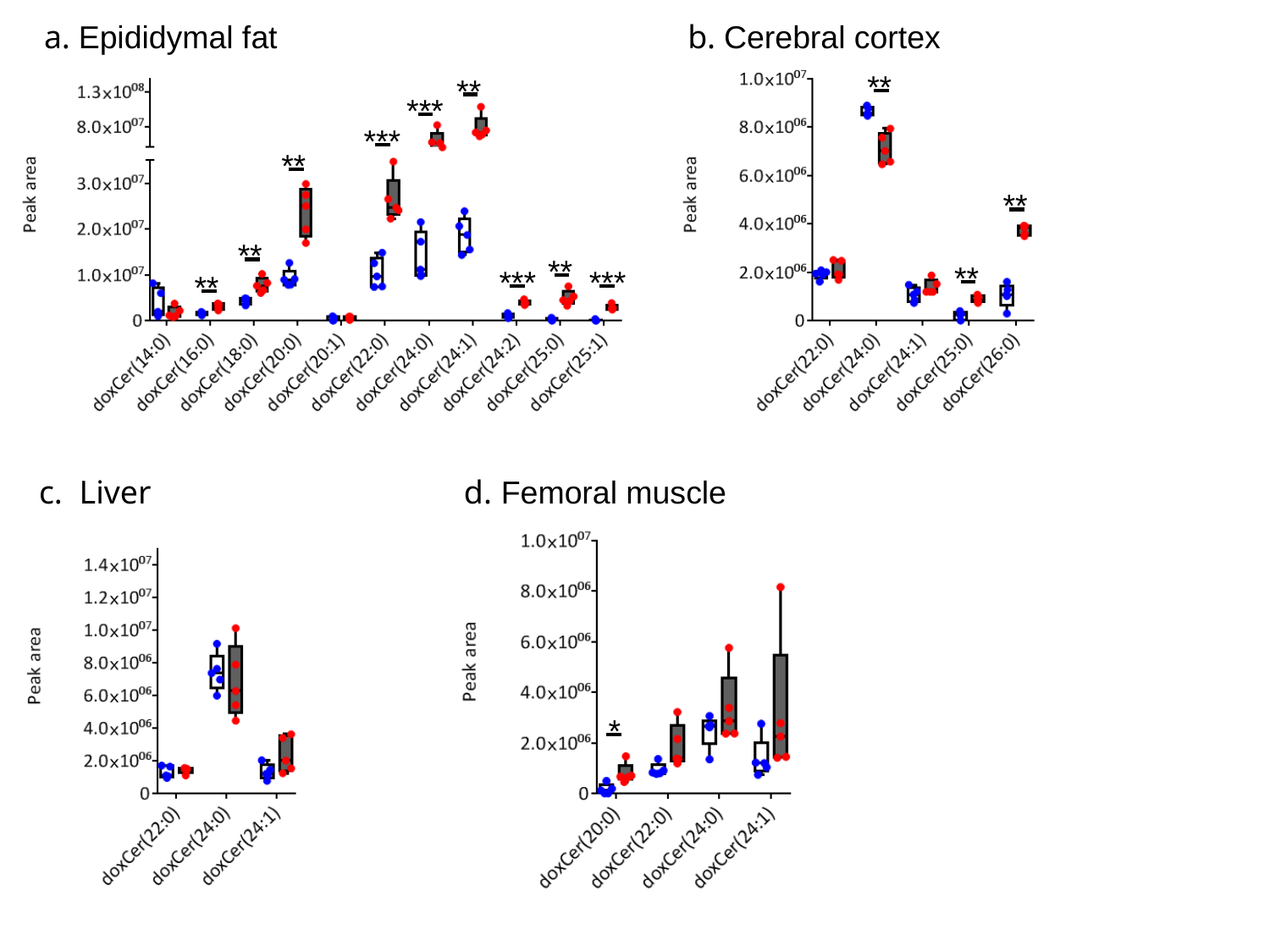

a. Epididymal fat 　 b. Cerebral cortex
**
**
***
***
**
**
**
**
**
***
***
**
c. Liver d. Femoral muscle
*

Supplement: Supplementary file 5 — Additional file 5. The doxCers in epididymal fat (a), the cerebral cortex (b), liver (c), and femoral muscle (d) in young (9 weeks old) and aged (114 weeks old) mice. Data are presented as box-and-whisker plot, with whiskers showing minimum and maximum values and each dot plot showing individual values (white bar and blue dot: young, gray bar and red dot: aged). P-values are indicated for the metabolites with significant differences in terms of abundance between groups. Abbreviations: DG, diacylglycerol; ae, acyl and ether. ***P < 0.001, **P < 0.01, *P < 0.05. [file 13578_2019_324_MOESM5_ESM.pptx]

## Slide 1
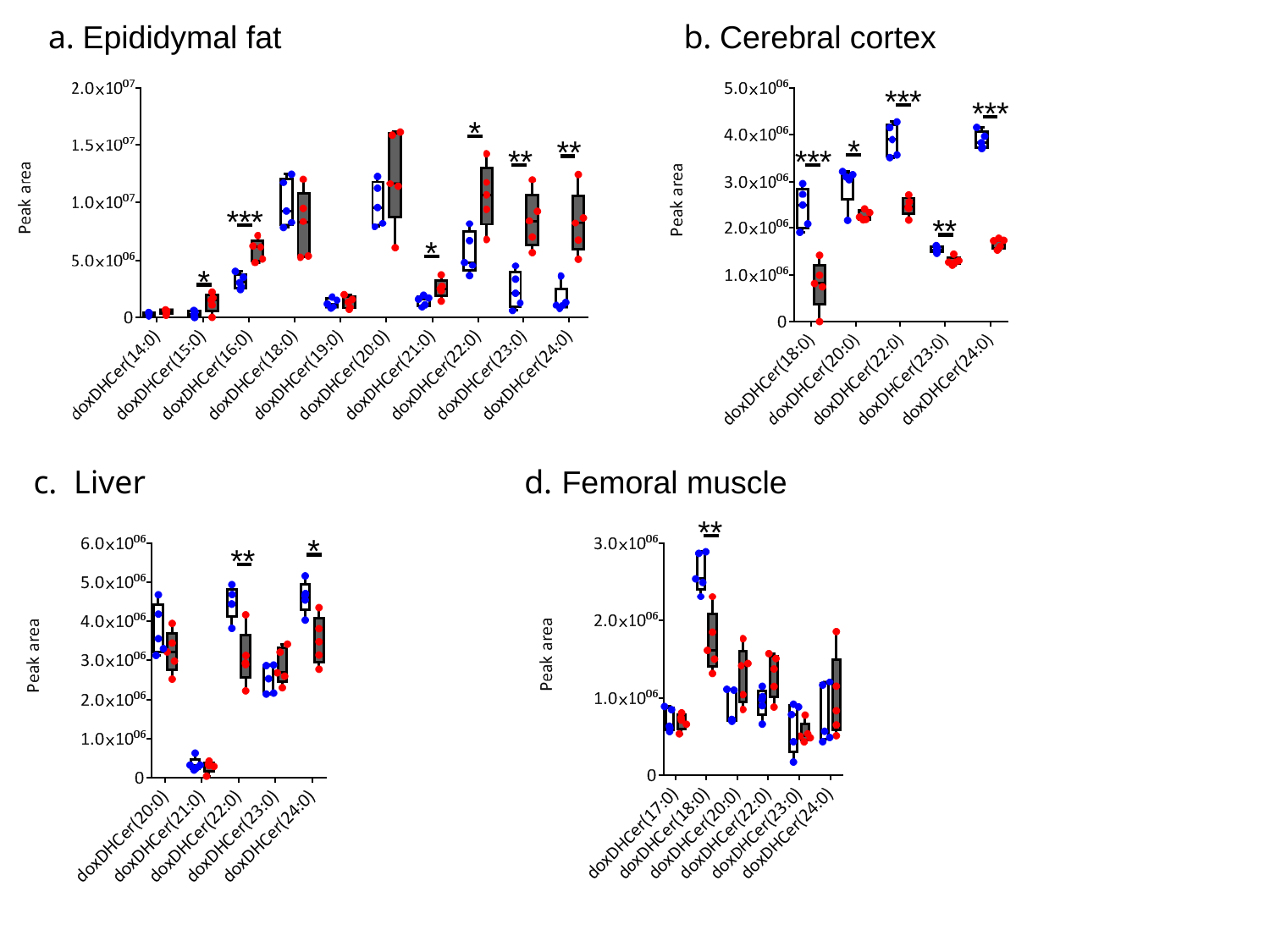

a. Epididymal fat 　 b. Cerebral cortex
***
***
*
*
**
**
***
***
**
*
*
c. Liver d. Femoral muscle
**
*
**

Supplement: Supplementary file 6 — Additional file 6. The doxDHCers in epididymal fat (a), the cerebral cortex (b), liver (c), and femoral muscle (d) in young (9 weeks old) and aged (114 weeks old) mice. Data are presented as box-and-whisker plot, with whiskers showing minimum and maximum values and each dot plot showing individual values (white bar and blue dot: young, gray bar and red dot: aged). P-values are indicated for the metabolites with significant differences in terms of abundance between groups. ***P < 0.001, **P < 0.01, *P < 0.05. [file 13578_2019_324_MOESM6_ESM.pptx]
